# Evaluating Costs Associated With Genetic Counseling Among Commercially Insured US Patients With Cancer From 2013 to 2019

**DOI:** 10.1001/jamahealthforum.2022.2260

**Published:** 2022-07-29

**Authors:** Mya L. Roberson, Nana Addo Padi-Adjirackor, Gillian Hooker, Tuya Pal

**Affiliations:** 1 Department of Health Policy, Vanderbilt University School of Medicine, Nashville, Tennessee; 2 Vanderbilt-Ingram Cancer Center, Nashville, Tennessee; 3 Concert Genetics, Nashville, Tennessee; 4 Department of Medicine, Vanderbilt University School of Medicine, Nashville, Tennessee

## Abstract

This cohort study describes the prevalence of out-of-pocket costs for cancer-related genetic counseling services in the US.

## Introduction

Genetic counseling by certified genetics professionals is an important part of the cancer treatment cascade for patients at risk for inherited sequence variants. Genetic counseling helps patients ascertain whether genetic testing is appropriate and helps in results interpretation.^1,2^ For some patients with pathogenic sequence variants, undergoing genetic testing affects treatment decisions and surveillance protocols in the survivorship phase.^3^ Coverage of genetic counseling varies across commercial insurers, and Medicare allows genetic counseling to be billed only under physician supervision.^1^ In this study, we aimed to identify total and out-of-pocket costs associated with genetic counseling in a US population of commercially insured adults with cancer and to describe factors associated with experiencing out-of-pocket costs for genetic counseling encounters.

## Methods

This cohort study was approved by the Vanderbilt University Medical Center Institutional Review Board, which waived the informed consent requirement because we used deidentified data. We followed the STROBE reporting guideline.

We used the IBM Watson Health MarketScan, a nationwide administrative claims database encompassing more than 30 million enrollees in large private insurance plans, to create a cohort of privately insured patients with breast, prostate, endometrial, ovarian, colorectal, or pancreatic cancer who had at least 1 encounter for genetic counseling between January 1, 2013, and December 31, 2019. These cancers have National Comprehensive Cancer Network guidelines for genetic or familial high-risk assessment.^4,5^ Outpatient genetic counseling encounters were identified using *Current Procedural Terminology* codes 96040 and S0265 among patients with 2 or more cancer diagnosis codes on 2 different days within the previous year. This method was used to identify individuals likely to have active cancer or to be cancer survivors and to reduce erroneous inclusion of codes for routine cancer screening.

We calculated out-of-pocket costs (sum of coinsurance, copayments, and deductibles) and total costs paid on claims for genetic counseling encounters. Using multivariable adjusted log-binomial regression, we calculated adjusted prevalence ratios (aPRs) for patients with out-of-pocket costs for genetic counseling services compared with those without out-of-pocket costs. SAS Studio (SAS Institute Inc) and α = .05 significance level were used in statistical analysis.

## Results

The cohort included 16 791 patients (15 570 women [92.7%], 1221 [7.3%] men), of whom 12 722 had breast, 1417 had colorectal, 1312 had ovarian, 622 had endometrial, 356 had prostate, and 312 had pancreatic cancer. Median (IQR) net payments for genetic counseling encounters were $118 ($58-$211) (Table 1). Most patients with cancer paid $0 for genetic counseling services, and the overall median (IQR) out-of-pocket cost was $0 ($0-$16). In total, 31.1% of patients had an out-of-pocket cost greater than $0. Patients billed under *Current Procedural Terminology* code S0265 had a lower prevalence of out-of-pocket costs than those billed under code 96040 (aPR, 0.52; 95% CI, 0.47-0.59) Table 2. Compared with patients with breast cancer, those with prostate cancer had a higher prevalence of experiencing out-of-pocket costs for genetic counseling (aPR, 1.28; 95% CI, 1.04-1.57).

**Table 1.** Characteristics of the Study Population for Initial Genetic Counseling Encounters by Cancer Type

Variable	No. (%)						
	All cancers (16 791)	Breast cancer (n = 12 772)	Ovarian cancer (n = 1312)	Prostate cancer (n = 356)	Endometrial cancer (n = 622)	Colorectal cancer (n = 1417)	Pancreatic cancer (n = 312)
**Age group, y**							
18-34	730 (4)	511 (4)	68 (5)	0	21 (3)	120 (8)	10 (3)
35-44	3211 (19)	2596 (20)	159 (12)	7 (2)	87 (14)	341 (24)	21 (7)
45-54	6682 (40)	5250 (41)	427 (33)	95 (27)	215 (35)	599 (42)	96 (31)
55-65	6169 (37)	4415 (35)	658 (50)	255 (72)	299 (48)	357 (25)	185 (59)
Genetic counseling procedure code							
96040	15 215 (91)	11 484 (90)	1218 (93)	331 (93)	577 (93)	1317 (93)	288 (92)
S0265	1576 (9)	1288 (10)	94 (7)	25 (7)	45 (7)	100 (7)	24 (8)
Billing provider							
Facility	9482 (56)	7230 (57)	754 (57)	199 (56)	341 (55)	803 (57)	155 (50)
Physician	6034 (36)	4568 (36)	460 (35)	137 (38)	222 (36)	519 (37)	128 (41)
Nonphysician^a^	1057 (6)	804 (6)	75 (6)	17 (5)	51 (8)	84 (6)	26 (8)
Other providers^b^	217 (1)	170 (1)	23 (2)	3 (1)	8 (1)	11 (1)	2 (1)
Encounter location							
Physician office	6903 (41)	5361 (42)	496 (38)	131 (37)	258 (41)	555 (39)	102 (33)
Hospital outpatient	9681 (58)	7263 (57)	802 (61)	216 (61)	356 (57)	842 (59)	202 (65)
Other setting^c^	203 (1)	148 (1)	14 (1)	8 (2)	8 (1)	20 (1)	5 (2)
Plan type, health^d^							
Non–high deductible	12 822 (76)	9723 (76)	1021 (78)	262 (74)	486 (78)	1103 (78)	227 (73)
High deductible	3663 (22)	2824 (22)	266 (20)	86 (24)	176 (28)	279 (20)	79 (25)
Data source							
Employer	12 265 (73)	9365 (73)	940 (72)	280 (79)	446 (72)	995 (70)	239 (77)
Health plan	4526 (27)	3407 (27)	372 (28)	76 (21)	176 (28)	422 (30)	73 (23)
Region^d^							
North East	3612 (22)	2727 (21)	346 (26)	60 (17)	112 (18)	286 (20)	71 (23)
North Central	3840 (23)	2900 (23)	287 (22)	83 (23)	159 (26)	330 (23)	81 (26)
South	5300 (32)	4114 (32)	339 (26)	110 (31)	198 (32)	452 (32)	87 (28)
West	3884 (23)	2918 (23)	322 (25)	98 (28)	143 (23)	332 (23)	71 (23)
Urbanicity, MSA^d^							
Within	14 811 (88)	11 292 (88)	1162 (89)	307 (86)	542 (87)	1233 (87)	269 (86)
Outside	1231 (7)	907 (7)	96 (7)	27 (8)	59 (9)	115 (8)	31 (10)
Cost, median (IQR), $							
Total	118 (58-211)	119 (58-213)	117 (60-198)	115 (55-211)	113 (54-201)	109 (53-199)	147 (65-235)
Out of pocket	0 (0-16)	0 (0-19)	0	0 (0-27)	0 (0-12)	0 (0-3)	0

Abbreviation: MSA, Metropolitan Statistical Area.

^a^ Nonphysician billing providers included advanced practice practitioners, including nurse practitioners and physician assistants.

^b^ Other billing providers included laboratories and public health agencies.

^c^ Other settings included independent laboratories, clinics, and telehealth.

^d^ Observations were missing for plan type (n = 306), region (n = 155), and MSA (n = 749). Urbanicity is defined as whether an enrollee resides within or outside of an MSA delineated by the US Office of Management and Budget.

**Table 2.** Proportion of Patients Experiencing Out-of-Pocket Costs for Genetic Counseling Encounters With Adjusted Prevalence Ratios for Out-of-Pocket Costs Greater Than $0

	Unadjusted proportion of patients with out-of-pocket costs >$0, %	Adjusted prevalence ratio (95% CI)^a^
Genetic counseling procedure code		
96040	32.3	1 [Reference]
S0265	19.5	0.52 (0.47-0.59)
Billing provider		
Physician	37.1	1 [Reference]
Facility	26.7	0.83 (0.78-0.90)
Nonphysician^b^	34.5	0.96 (0.87-1.04)
Other providers^c^	37.2	1.01 (0.84-1.21)
Encounter location		
Physician office	34.8	1 [Reference]
Hospital outpatient	28.3	0.86 (0.80-0.92)
Other setting^d^	35.5	0.94 (0.77-1.14)
Cancer type		
Breast	32.8	1 [Reference]
Colon	25.1	0.78 (0.69-0.88)
Endometrial	28.1	0.84 (0.74-0.96)
Ovarian	23.3	0.73 (0.66-0.81)
Pancreatic	16.7	0.53 (0.41-0.69)
Prostate	38.5	1.28 (1.04-1.57)
Plan type, health		
Non–high deductible	31.7	1 [Reference]
High deductible	29.3	0.87 (0.82-0.92)
Data source		
Employer	32.3	1 [Reference]
Health plan	27.8	0.84 (0.80-0.89)

^a^ Adjusted for procedure code, billing provider, encounter location, cancer type, plan type, data source, age, sex, region, and urbanicity (defined as whether an enrollee resides within or outside of a Metropolitan Statistical Area delineated by the US Office of Management and Budget). Age, sex, region, and urbanicity were not associated with cost in adjusted models.

^b^ Nonphysician billing providers included advanced practice practitioners, including nurse practitioners and physician assistants.

^c^ Other billing providers included laboratories and public health agencies.

^d^ Other settings included independent laboratories, clinics, and telehealth.

## Discussion

Cancer genetic counseling not only promotes informed decision-making about genetic testing and cancer treatment in the era of precision medicine but is also a form of low-cost, high-value care.^6^ More frequent out-of-pocket costs for patients with prostate cancer may reflect a lack of awareness about the medical necessity of genetic counseling, a disservice given the recent inclusion of aggressive or metastatic prostate cancer in National Comprehensive Cancer Network guidelines.

Study limitations include lack of clinical information such as cancer stage at diagnosis and clinical subtype, which could support the receipt of and insurance payment for genetic counseling. Furthermore, some genetic counseling encounters are not billed for, likely representing the undermeasurement of total service utilization. Although costs for patients were low, because the Centers for Medicare & Medicaid Services does not recognize certified genetic counselors as a billable providers, genetic counciling costs may be shifted to health care practices. The findings highlight the relatively low financial costs of genetic counseling, a form of care with potentially substantial implications for cancer treatment.

## References

[1] Harrison TA, Doyle DL, McGowan C, . Billing for medical genetics and genetic counseling services: a national survey. J Genet Couns. 2010;19(1):38-43. doi:10.1007/s10897-009-9249-5 19809868

[2] Hallquist MLG, Tricou EP, Hallquist MN, . Positive impact of genetic counseling assistants on genetic counseling efficiency, patient volume, and cost in a cancer genetics clinic. Genet Med. 2020;22(8):1348-1354. doi:10.1038/s41436-020-0797-2 32350418

[3] Riley BD, Culver JO, Skrzynia C, . Essential elements of genetic cancer risk assessment, counseling, and testing: updated recommendations of the National Society of Genetic Counselors. J Genet Couns. 2012;21(2):151-161. doi:10.1007/s10897-011-9462-x 22134580

[4] Creasman WT, Berry LK, Talavera F, et al. What are the NCCN joint guidelines for genetic testing of women with endometrial carcinoma? Accessed October 3, 2021. https://www.medscape.com/answers/254083-116433/what-are-the-nccn-joint-guidelines-for-genetic-testing-of-women-with-endometrial-carcinoma

[5] Daly MB, Pal T, Berry MP, . Genetic/familial high-risk assessment: breast, ovarian, and pancreatic, version 2.2021, NCCN Clinical Practice Guidelines in Oncology. J Natl Compr Canc Netw. 2021;19(1):77-102. doi:10.6004/jnccn.2021.0001 33406487

[6] Stoll K, Kubendran S, Cohen SA. The past, present and future of service delivery in genetic counseling: keeping up in the era of precision medicine. Am J Med Genet C Semin Med Genet. 2018;178(1):24-37. doi:10.1002/ajmg.c.31602 29512888

